# Women’s expectations about birth, requests for pain relief in labor and the subsequent development of birth dissonance and trauma

**DOI:** 10.1186/s12884-023-06066-7

**Published:** 2023-11-09

**Authors:** Elizabeth Sutton, Karen Detering, Christine East, Andrea Whittaker

**Affiliations:** 1 Monash Bioethics Centre – Monash University, Melbourne, Australia; 2 Department of Health and Aged Care, Melbourne, Australia; 3 Nursing and Midwifery – La Trobe University, Melbourne, Australia; 4 Sociology and Anthropology – Monash University, Melbourne, Australia

**Keywords:** Women, Labor, Pain management, Natural childbirth, Normal childbirth, Midwifery, Obstetrics, Request, Dissonance (cognitive)

## Abstract

**Background:**

Birth is a significant event in women’s lives. As Mansfield notes (2008) many women aim for a birth that avoids pharmacological pain relief because they are advised it is better for them and their baby. For women having their first baby, this may not be realistic as 3/4 of primiparous women in Australia will use pharmacological pain relief. This study examines the expectations that a group of women had regarding pain relief, how these expectations developed and what happened to requests for pain relief in labour.

**Methods:**

A longitudinal prospective study design was used to recruit 15 women who were having their first baby. Women having low risk pregnancies, hoping for a ‘natural birth’ (vaginal, no/minimal pharmacological pain relief) were eligible. A semi-structured interview tool was used across all three interviews that asked women about their expectations, then actual labour experience, pain management requests and how these were responded to by carers. Fifteen women were interviewed – at 36 weeks gestation; as soon after delivery of their baby as possible, then six months post-delivery (*N* = 43 interviews). Interviews were recorded and transcribed and coded by ES using NVivo software with hierarchical thematic analysis used.

**Results:**

The study found that women appear to experience a mismatch between expectations they had developed pre-birth, versus actual experience. This appears to cause a specific form of dissonance – which we have termed ‘birth dissonance’ leaving them feeling traumatised post birth. This is because what women expected to happen in birth was often not realised. In particular, some women requested pain relief in birth and felt that their request was not responded to as hoped, and also seemed to develop post-birth trauma. We proposed that this may have resulted from dissonance arising from their expectations about being able to birth without significant pain relief. Interventions and technology may also contribute to this sense of mismatch and post-birth trauma.

**Conclusions:**

Low risk birthing women birthing in a hospital may have to engage with higher levels of technology, intervention and pain relief than that which they expected pre-birth. This could possibly be avoided with four simple changes. Firstly, better pre-birth education for women about how painful labor is likely to be. Secondly, pre-birth education which includes a detailed explanation of the utility of pharmacological and non-pharmacological pain relief. Thirdly, more egalitarian decision-making during labour and finally delivering upon women’s requests for pain relief in labor, at the time that they ask for it. Further research is required to determine the extent of birth dissonance and how women making the transition to motherhood can avoid it.

**Supplementary Information:**

The online version contains supplementary material available at 10.1186/s12884-023-06066-7.

## Introduction

Childbirth may be a painful experience for women hoping to have a baby vaginally without pain relief – especially for first births. As in the UK, Canada and parts of the United States, nearly all Australian women (96%) deliver their baby in a hospital [1] and thus have access to the full formulary of pain relief. Many women, however, for their first birth, aim for a vaginal birth without pharmacological pain relief [2] – often referred to as ‘natural birth’[3]. Normal and natural birth are contested terms, confusingly interchanged across the literature [4–7]. For the purposes of this article, natural birth refers to a vaginal birth with no pharmacological pain relief used and with non-pharmacological pain relief options described below. By contrast, so called normal births can be vaginal births that may or may not involve the use of pharmacological pain relief. Hospital based maternity services in Australia aspire, to greater or lesser extents, to provide a range of choice of woman-centred care during pregnancy, labor, birth and post-natally thus encompassing preferences for a ‘natural’ or ‘normal’ birth [1].

Non-pharmaceutical analgesia may include movement, warm water, breathing techniques aromatherapy and massage, many of which lack high quality evidence to support their efficacy [8–10]. This may or may not be of concern if women find them useful and as complementary in the overall labor experience [11]. For example, techniques that result in relaxation, such as hypnosis, breathing techniques, music, massage, may contribute to the woman’s mindful sense of managing with the pain and/or able to continue to be an active contributor in leading choices about how the labor and birth proceed [10, 12].

Pharmaceutical interventions have been subjected to numerous randomized controlled trials and other studies [13–15]. Many women aspire to achieve natural births, without wanting to use other readily available, effective and safe pain relief methods [16, 17]. A Cochrane review has found no evidence suggesting differences in rates of instrumental birth and cesarean between women having an epidural compared to those not having one (and/or other pain relief interventions), when studies post 2005were reviewed [13]. The only statistically significant difference found in the post 2005 studies in outcome was that the first and second stages of labor were longer by approximately half an hour for women with an epidural; this may or may not be viewed as clinically important by women or their caregivers. Women with an epidural required less pain relief for breakthrough pain [13]. For all other measures, it found that women do not have higher rates of instrumental birth, cesarean section, long term backache, nor were there differences in neonatal Apgar scores or rates of admission of babies to neonatal intensive care [13]. This means that the ‘cascade of intervention’ [18, 19] associated with earlier forms of epidurals appears to have been overcome with a combination of greater nuance and finesse in drug dosage, and possibly improved placement by anaesthetists and better equipment [20].

The experience of childbirth, however, is different to other pain related events [21]. Pain in labor is classified as an acute pain. This pain may be viewed from perspectives along a continuum of polar opposites, through to those which are complementary. These include, but are not limited to, women being passive recipients of care viewing pain as purposeful and productive [22], an important part of becoming a mother [23] and/or liberally applying pharmaceutical methods of analgesia to minimize or eliminate pain. These all need to be considered as they form part of the milieu in which a woman will birth, and thus may also influence her ability to cope. For some women, the actual social meaning ascribed to the experience of laboring leads them to aim for vaginal, drug free births in the belief that birthing this way is better for themselves and their baby, and that women are ‘designed’ for birthing [6, 7].

Women may also have been educated that birthing without pharmacological pain relief and vaginally, or that vaginal birth post cesarean, can be a transformative event [5, 24, 25]. The term ‘technocratic birth’ [25] is a linguistic summary of the combined technological and bureaucratic management of birth and it is argued that labor has become more a medical experience than a social one. Pre-birth classes and popular text-books on birth [26, 27], and various internet forums, pages and sites discuss vaginal birth with no pain relief as being best for both mothers and babies [23, 26]. These classes, books and internet sites (see for example: https://mamanaturalbirth.com) often encourage women to avoid pharmacological pain relief so that they can avoid complications from pain relief, but also come to understand the power of their bodies in new ways [28, 29]. Sanders and Crozier note that women do not enter the birth experience as ‘empty vessels’ but rather have usually gathered knowledge from various sources [30]. It is therefore important that women are aware that the knowledge that they hold may not necessarily reflect what could happen to them during labor. This is because, as we argue, a fixedness on how their birth might unfold, is a likely cause of cognitive dissonance, causing distress and possibly leading to PTSD [31] and other post birth trauma.

While some women whose good birth experiences also include being able to work with their pain and/or minimal pain relief, others find the pain unbearable, disempowering and even traumatising [32, 33]. This can certainly be the case when birth does not go as planned [34, 35]. Birth trauma is an increasingly recognised phenomenon [32, 35, 36],and there is now a validated tool to measure birth trauma, indicating its increasing acceptance as an outcome post-delivery [37]. We argue in this paper that women can develop birth trauma because they have experienced a phenomenon, or ‘birth dissonance’. Dissonance is a well-established psychological theory published originally by Festinger [38] and recently expanded upon by other scholars [29]. It is an important concept here because it is argued to arise when there is intellectual inconsistency that becomes internalised In this instance, we suggest that birth dissonance arises when women experience a fundamental disparity between what they had expected of birth and their body, and what actually occurs [36].

There has been growing recognition that for some women their experience is not just traumatic, but rather, closer to ‘violence’ and the term obstetric violence [39–42] is now used to describe this experience – although it should be noted it does have particularities associated with it. This term applies where unwanted, invasive and painful procedures are visited upon women with little or no consultation (and thus dubious consent), leading to women feeling violated. It has its origins in the Southern Americas and is closely associated with medical care where scope of practice enables more invasive and complete violation of the self under the guise of delivering lifesaving ‘care’, but midwifery is not immune from criticisms [37, 39]. The birth trauma we discuss here is not a form of obstetric violence per se, mostly because women being asked to delay receipt of pain relief are not being physically accosted. While the women in this study may have had their autonomy impinged upon by virtue of the request not being met, this is not the same as the intentional action of someone who uses instruments to assert their power over a vulnerable patient.

While most health professionals clearly mean to do their best by women, it appears that they can inadvertently contribute to women experiencing birth trauma. Such trauma may have multiple causes and may even appear when health care professionals consider the birth to have been relatively routine; indeed women report mixed emotions about future births when asked about them by Rilby and colleagues [43]. Birth trauma may arise because women have more intervention than anticipated, but can extend to laboring women fearing for their lives or those of their unborn child/ren. Also having pain during birth which needs pharmacological treatment and is not treated, and finally the overall subsequent mismatch between reality and their expectations should this occur [44–46] can lead to post birth trauma.

One of the key causal factors resulting in birth trauma is intense pain or discomfort [42] that can lead to suffering. Given that pain relief is available to most birthing women in Australia, this seems a preventable cause of birth trauma. Nearly all women in Australia birth in a hospital with access to the full formulary of pain relief, as well as usually having midwifery support, medical care and anaesthetics available, with low rates of fetal and maternal mortality [47]. In spite of this, and the best efforts of staff to support women during birth, a proportion of women continue to report birth to be a traumatic event. Studies conducted in Australia and similar countries have found that somewhere between one sixth to one third of these women will consider birth to be a traumatic event [29–31] even though the outcome is usually a live mother and baby. It appears that even if birth results in a live baby and live mother, it can still cause great harm.

Pain relief usage from the Australian Institute of Health and Welfare (AIHW) data show that in 2020, 79% of women who experienced labor, had some type of pharmacological pain relief. The most common types of pain relief used were nitrous oxide (used by 51% of laboring women), regional anaesthetic (used by 40% of laboring women) and systemic opioids (used by 12% of laboring women) [47]. One fifth of women (20.5%) did not use any pain relief [47]. Some women used more than one type of analgesia. The use of pain relief in labor when a woman has prepared for vaginal, drug free birth, appears to be a source of a particular type of trauma for women, even when the outcome is good [46]. This may be because their expectations regarding how birth would unfold, is not what actually happened [32, 33, 36, 48–52].

This study investigated the way a group of women planned for a vaginal birth. Using research questions that were applied to the development of the interview tool/s, the study examined the various pain relief options that women expected to use during their impending births; how they experienced birth; and if their plans for their chosen pain relief options were realised. The study also sought to ascertain if laboring women asked for types of pain relief that they had been hoping to avoid, and how these requests for pain relief were responded to by carers. Finally, women were asked about their birth experience and whether the expectations that they had prior to birth about pain and pain management were reasonable, given their lived experience of birth. The key research foci for this study revolved around how women developed expectations about pain relief in labour and how requests for analgesia were met. An emergent question as the analysis of the post-birth interviews proceeded was how their experiences may or may not have negatively affected their psychological well-being.

## Methods

This qualitative study used a case series design and a limited longitudinal approach. It was designed by a multidisciplinary team comprised of a post-graduate medical anthropology candidate, a sociologist, an anthropologist and an academic midwife with previous lived midwifery experience. Additional advice was provided from a senior anaesthetist, and a consulting physician involved in advance care planning. A convenience sample of nulliparous women was recruited from a single site, from a specifically chosen low risk ‘outpatients’ clinic at a large public hospital in Melbourne, Australia. The data reported in this paper form part of a more extensive study on pain relief during birth which involved interviewing different health care professionals (obstetricians, midwives and anaesthetists) as well as primiparous women who were nulliparous at the study commencement. This paper relates only to the data provided by women expecting their first baby.

Women were recruited and once consented, were interviewed three times over an 8-month period. The interviews took place at three time points: approximately at 36 weeks’ gestation; once as soon after the birth of their baby as possible; and then six months later. A total of 43 interviews were conducted with 15 (initially nulliparous) women as part of this study.

The maternity service where women were recruited from for this study was primarily midwifery led. Trainee obstetricians (both residents and registrars) were rostered on and on-call throughout the 24-h period every day. Consultants were on call and available for more complicated deliveries. Anaesthetists were on call at all times as they serviced the entire hospital and not just the labor ward/birthing unit. Women had access to movement, warm water, aromatherapy (if they brought it in themselves) birthing balls, TENS machines ( if self-obtained), hypnosis if trained, nitrous oxide and oxygen, intramuscular opioids; sterile water injections (for back pain), epidurals, spinal blocks, and general anaesthesia if necessary.

Ethics approval for the study was provided by the Monash Health Human Research Ethics Committee. All research and interviews were conducted in accordance with relevant guidelines [53] and informed consent was obtained from all participants. Participants could only participate if they were able to provide own written informed consent (no guardian/carer arrangements); and therefore were over the age of 18 and also willing to be involved. Inclusion criteria were women who had had no previous experience of birth or labor, had an intention to try and have a ‘natural’ (vaginal birth as described above) birth, were low risk, singleton pregnant women, aged 18 – 35 years, were willing to be involved in the study. As noted above, they had to be able to give their consent (no agents/guardian acting on their behalf). Women had to be able to speak, read and understand English as interpreter services for the study were not available. Attachment A contains the interview questions for women both pre and post birth.

In pre-birth interviews, women were asked a series of demographic question, then open-response questions to prompt them to talk about how they had developed their plan for their birth, what expectations they had regarding the management of their birth, the length of labor, how they would manage pain during labor and how they had developed these expectations. After labor and birth, women were asked similar questions with the addition of questions about how they felt their pain was managed and if they had asked for pain relief what happened to their request and how it was responded to by their carer/s. If women started to talk about issues of trauma following their birthing experience, the interviewer explored this to ascertain information about mental health services that they had used; if they had experienced post-traumatic stress disorder type symptoms as described by them, had been hospitalised, required medication; or sought other formal, medical or community care based interventions for their emotional well-being post birth over and above the usual services provided to women post birth.^1^ Women who reported themselves as being upset or distressed, but who had not engaged formal mental health services or medication, or were not delaying future children, were not regarded as suffering post-birth trauma for this study.

Interviews were transcribed by ES, and then coded with NVivo software to identify key themes with subthemes also being coded using hierarchical node analysis in NVivo QSR Intl. Grounded theory was used to determine the key issues that needed re-clarification or re-presentation at second and third interviews. A linguistic content approach was used alongside grounded theory to better understand terms that were used frequently, and often in variable ways across participants such as ‘normal birth’; ‘natural birth’; pain relief; and ‘asking’ for pain relief. In some instances, where women appeared to be particularly traumatised, their medical record was reviewed to determine what had been written about their experience, versus their recollection. This was undertaken with their permission which was collected at the time of consent for involvement in the study and with HREC approval.

## Results

Forty-five women initially agreed to be in the study but as their pregnancies progressed, a number of women developed complications such as pre-eclampsia, gestational diabetes, were induced early, some moved away from the study area, and others declined follow up calls. As a result, 15 women were eligible for inclusion of which 13 were interviewed three times and 2 were interviewed twice. These interviews occurred at approximately 36 weeks’ gestation, next as soon after delivery as possible, and for a third time at approximately six months after delivery. As noted above, one woman’s pre-delivery interview did not occur, and one woman did not complete the final interview at the six-month period despite follow ups. In all, 43 interviews were completed and used for analysis.

Participants were all aged between 22 and 32, one had completed high school, all others had additional qualifications. Their occupations included teaching, nursing, administration, engineering and occupational therapy. All were living in domestic partnerships and with one exception, all were employed at the time that they were recruited for this study. All names have been changed and pseudonyms have been assigned to women in the reporting that follows.

### Birth that women wanted – pre-labor interview results

The results from the pre-labor interviews are summarised in Table 1 (overleaf) which documents what women in the study wanted to use for their pain management prior to labor, and what they wished to avoid.

**Table 1:** Pre labor-interview results with nulliparous women regarding birth wishes and pain relief methods

**Name**	**Expected length labor**	**Women’s wishes pre-labor – pain relief methods**	
		**Will use**	**Wants to avoid**
*Yvonne*	8 – 11 h	Meditation, warm water, movements, TENS^a^, aromatherapy, nitrous oxide, water injection	Hypnosis, deep tissue massage, pethidine, epidural and spinal block but will use if necessary
*Andrea*	24 or longer	Warm water, movement, TENS, nitrous oxide, pethidine, epidural	Meditation, hypnosis, deep tissue massage, aromatherapy, spinal block, other
*Indigo*	24 – longer	Warm water, nitrous oxide, epidural	Meditation, hypnosis, TENS, deep tissue massage, aromatherapy, spinal block, other
*Narelle*	24 or longer – not sure	Nitrous oxide, pethidine; keen to avoid epidural but will use it if necessary	Meditation, hypnosis, warm water, deep tissue massage, TENS, aromatherapy
*Linda*	12–15 h	Warm water, DTM^b^, movement, paracetamol, nitrous oxide if needed keen to avoid	Meditation, hypnosis, TENS, pethidine, epidural, spinal block
*Hannah*	8- 11 h	Warm water, DTM, TENS, nitrous oxide, pethidine	Meditation, hypnosis, not aware of epidural or spinal block
*Yolande*	12 – 15 h	Warm water, movement, nitrous oxide	Meditation, hypnosis, DTM, TENS, pethidine, epidural, spinal block
*Naeve*	20-23 h	Warm water, movement, TENS, nitrous oxide	Aromatherapy, meditation, pethidine, hypnosis, deep tissue massage, epidural, spinal block
*Eleanore*	24 h – hoping no more	Heat packs, music, hot water, movement, nitrous oxide	Aromatherapy, meditation, hypnosis, TENS, pethidine, epidural
*Lee-Ann*	24 – 27 h – hoping no more	Nitrous oxide, epidural	Aromatherapy, meditation, hypnosis, TENS, pethidine
*Louise*	18 h	Nitrous oxide, meditation, warm water, movement	Hypnosis, TENS, pethidine, epidural, spinal block
*Yve*^d^	12–15 h	All methods considered if necessary	Epidural
*Haley*^c^	8 h	Movement, nitrous oxide, epidural	Meditation, hypnosis, Deep tissue massage. Not aware of pethidine or other opiates being available or TENS
*Deliah*	18 h	Movement, Pethidine	Hypnosis, warm water, DTM, TENS, aromatherapy, nitrous oxide, epidural
*Yasmin*	4 h	Heat packs, warm water, massage, movement, nitrous oxide	Meditation, hypnosis, aromatherapy, epidural, pethidine

^a^TENS Transcutaneous electrical nerve stimulation

^b^Deep Tissue Massage

^c^Pre Labor wishes collected post-labor event

^d^did not complete 6 month follow up interview

All women who participated in the study indicated that they were hoping to have a vaginal birth and avoid pharmacological pain relief if possible. Some women were clear at the outset that they would engage pharmacological pain relief if necessary and the form of this pain relief was most commonly nitrous oxide. Fourteen of the 15 women indicated that they wished to use this if their non-pharmacological pain relief methods did not provide adequate relief. Ten of the 15 women indicated a clear wish to avoid an epidural. None of the women contemplated a surgical birth or conversely, a birth requiring forceps or other high-level intervention to enable vaginal delivery.

### Birth that women experienced – post-labor interview results

Of the 15 women in this study, all asked for some type of pharmacological pain relief. Only one woman labored with the type of pain relief and had the type of birth she expected – Naeve (marked by^^ below). All other women engaged technology and/or pain relief over and above what they expected. All other methods in listed above were used by various women (Table 2).

**Table 2:** Post-labor interview results with women regarding birth wishes and pain relief methods desired pre-birth with post-birth outcomes including birth dissonance^a^

Name	Expected length labor	Actual Labor length	Women’s wishes pre-labor – pain relief methods Will use	Type of delivery AND Unforeseen interventions/technology/pain relief used in red	Pain relief requested/needed = Y/N Pain relief request met = Immediately, Deferred or Never met	Trauma = mental health services/medication/Hospitali-sation/tokophobia
*Yvonne* BD^a^	8 – 11 h	21 h	Meditation, warm water, movements, TENS, aromatherapy, nitrous oxide, water injection	Vaginal delivery **Neville Barnes forceps;** Late pudendal block – no spinal block Movement, warm water, TENS, nitrous oxide	Yes *Never met– reported pain as very badly managed –felt ignored by staff*	Yes
*Andrea* *BD*^a^	24 h or longer	22 h	Warm water, movement, TENS, nitrous oxide, pethidine, epidural	Vaginal delivery **forceps delivery;** Movement, nitrous oxide, TENS, epidural,	Yes *Deferred by midwifery staff – then provided several hours later*	Yes
*Indigo* *BD*^a^	24 h or longer	6 h	Warm water, nitrous oxide, epidural	Vaginal delivery **syntocinon** Movement, nitrous oxide	Yes *Never met– told not required by midwifery staff*	Yes
*Narelle* *BD*^a^	24 h or longer – not sure	12 h	Nitrous oxide, pethidine; keen to avoid epidural but will use it if necessary	Vaginal delivery **forceps** Nitrous oxide, pethidine, **spinal block**	Yes Immediately	Yes
*Linda*	12–15 h	7.5 h	Warm water, DTM, movement, paracetamol, nitrous oxide if needed keen to avoid	**Surgical delivery – Emergency caesarean, epidural** Paracetamol, warm water, gas, DTM, movement, nitrous oxide,	Yes *Deferred – owing to staff availability then met*	Yes
*Hannah*	8- 11 h	6 h	Warm water, DTM, TENS, nitrous oxide, pethidine	Vaginal delivery **Induction for H/T- Syntocinon** Nitrous oxide	Yes Immediately	No
*Yolande*	12 – 15 h	15 h	Warm water, movement, nitrous oxide	Vaginal delivery **Epidural in place** Warm water, heat pack, massage, movement, nitrous oxide, epidural	Yes Immediately	No
*Naeve^^*	20-23 h	24 h	Warm water, movement, TENS, nitrous oxide	Vaginal delivery Movement, TENS, Nitrous oxide	Yes Immediately	No
*Eleanore*	24 h – hoping no more	22 h	Heat packs, music, hot water, movement, nitrous oxide	Vaginal delivery Movement, nitrous oxide, **epidural (failed)**	Yes Immediately	No
*Lee-Ann*	24 – 27 h – hoping no more	14 h	Nitrous oxide, epidural	**Surgical Delivery** **Caesarean post admission** **Syntocinon for contractions** Nitrous oxide, epidural	Yes Immediately	No
*Louise*	18 h	17.5 h	Nitrous oxide, meditation, warm water, movement	Vaginal delivery Nitrous oxide, warm water, **epidural**	Yes *Deferred – then met*	Yes
*Yve*	12–15 h	7 h	All methods considered if necessary	Vaginal delivery Nitrous oxide, **epidural**	Yes *Deferred – then met*	No
*Haley* *BD*^a^	8 h	22 h	Movement, nitrous oxide, epidural	Vaginal delivery **meconium in waters, syntoconin for contractions, face presentation, partially retained placenta, small PPH** Movement, gas, **sterile water injections**	Yes *Never met*	Yes
*Deliah*	18 h	24 h	Movement, Pethidine	Vaginal delivery **posterior presentation** Warm water, **nitrous oxide,** pethidine, **sterile water injections**	Yes *Deferred – then met*	No
*Yasmin* *BD*^a^	4 h	1.5 h	Heat packs, warm water, massage, movement, nitrous oxide	Vaginal delivery nitrous oxide **Precipitous labor, ruptured membranes at 33 weeks and 6 days, delivered 34 weeks and 1 day**	Yes *Never met – (inadequate time)*	Yes

^a^Birth Dissonance

*^^*Only woman in the study who labored with the type of pain relief she had hoped to use and had the birth she expected

Post-labor, women appeared to have very good recall of the events as they occurred. A number of these women reported that they felt that they had not done birth ‘properly’, or even ‘failed’. Deferral or denial of pain relief requests or pain that was ignored altogether appeared to result in greater trauma. This has been conceptualised in this paper as a specific form of dissonance – namely ‘birth dissonance’—and needed specific treatment.

**Fig. 1:**
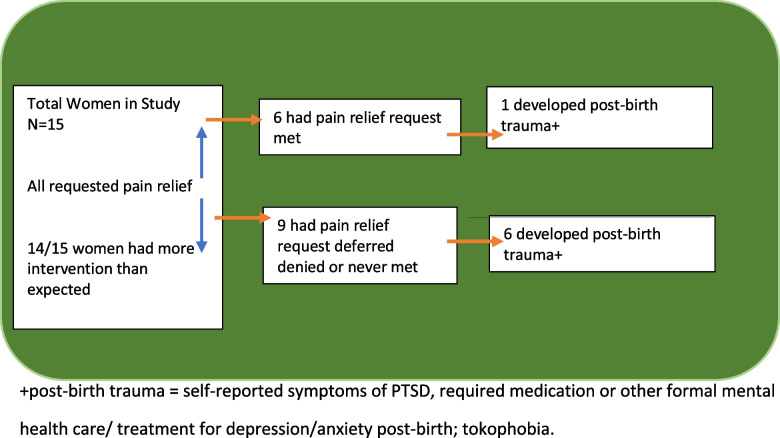
Pain relief requests, responses, and development of post-birth trauma

Six of the 15 women reported their pain relief outcomes as being met at first request or considered their pain well managed. The remaining nine women described their pain relief request as either deferred and then met, or never met. Of these nine, six women (Yvonne, Andrea, Indigo, Linda, Haley and Yasmin) had higher levels of intervention in their birth than anticipated or had a delivery that was pre-term, or involved the application of forceps to assist delivery. Six of these nine women also reported post-birth trauma that required treatment via therapy/counselling, medication, in-patient hospital stays or resulted in the development of tokophobia. Linda and Louise did not require treatment but were disappointed in their births at the first post-birth interview and Linda was still tearful about it six months later. In short, women who went on to develop post-birth trauma symptoms were likely to not have their pain relief request met or immediately met (Fig. 1).

Importantly, some women (see Yve and Deliah) did not develop post birth trauma symptoms as per the definition used in this paper (see: page 10) even though their pain relief request was deferred. That is, they did not require GP consultation, medication for post birth anxiety, hospitalisation or psychogical support over and above usual services for women who have delivered a baby. For the six women in this study who did have their pain relief request responded to once made, one developed post-birth trauma type symptoms. This suggests that there are multiple factors at play in birth, and that pain relief being needed is just one element – albeit an important one—of the multifaceted experience of childbirth that may be traumatising.

#### Building expectations of the ability to give birth ‘naturally’

As part of this study, women were asked about why they were hoping to have a low intervention, generally drug-free labor to consider how women developed their expectations about pain management, prior to labor. As Yvonne notes:

*"…it seems to just be…. I don’t know, an expectation, a pressure. People just want to know did you have a natural birth...it's one of the first questions you get asked, did you do it yourself, and did you do it naturally ….I guess it's those sorts of stories where it puts pressure on you and you think ‘Oh well, why can't I do it?’ (Yvonne, Int 3)*

For Yvonne, it is the assumption of the broad availability of a vaginal birth that means that she expected to be like other women who were able to birth vaginally and without intervention. Wanting to know about how you birthed, appears to then be used as a barometer of the womanliness of each woman and leaves women wondering about their womanliness if they could not birth ‘naturally’.

Linda also notes that there is a ubiquitous and broadly held expectation that women will birth ‘naturally’. After Linda spoke of how her plans for a natural, drug free, vaginal birth were set aside and she had an emergency cesarean owing to fetal distress, she was asked why she thought women felt this pressure to have babies ‘naturally’. She responded:

*….I see the stories, I see people posting things and saying that someone said to them - you didn't give birth - you took the easy way out…you did this, because you had an emergency c section or a c section for whatever reason and I feel that it seems that a lot of people think that's how you should give birth in a natural way in a natural environment….It's one of those things that comes down to society again, again its what's expected of you, it's the normal way to do things. …I thought you know well if everyone can do it I can do it. (Linda, Int 2)*

Again, there is not a specific source from where these expectations are generated, but rather they arise from the broader birthing milieu in Australia in which women are socialised on a daily basis. This is added to by high profile public figures who praise their partners on social media for being ‘so brave’ for not using pain relief while declaring they are so ‘proud of her’ (harrykane, Instagram 8 August 2018). These assertions buttress a mythology that a woman’s goodness is evidenced by her willingness to suffer for the betterment of both herself and her baby, influencing women as they prepare for labor.

#### Asking for pain relief in labor

Having developed a plan for labor that included avoiding pharmacological pain relief, a number of women found that they had to depart from that plan during labor because of either the length of the labor, the pain in labor being greater than what they expected, or both. Women were asked “what happened when they asked for pain relief – how was the request responded to by their health care professional?”

Indigo responds to this question the following way:

*"Umm, I think they said that you are coping really well, and you don't need one".*

*Int: How did they know that, did they ask you how your pain was?*

*"No, I think just because I wasn't a screamer, I sort of go into myself when I have pain, so they just said they would turn up the gas and see how you go….another twenty minutes and see how you go with that… (Indigo, Int 2).*

Indigo gave birth relatively quickly after this request, however she had made the request twice and had also been given oxytocin (syntocinon) to speed her labor.

Another woman Louise – talks about how her pain relief request for an epidural was deferred and she then entreats her partner to act on her behalf. She says that after a number of refusals for an epidural, she tells her carers:

*I said, “Nup, you guys need to do this [epidural]”. And I just kept saying to my partner, “You need to make them do it….I was like I don't care about that woman [midwife], it's making me really angry”. I was “No, I don't care, she needs to [organise an epidural]”, because she was pretty active in trying to convince me otherwise….(Louise, Int 2).*

Louise’s initial requests were not met, so in order to strengthen her position, she asked her partner to advocate on her behalf. The description of a midwife who was “pretty active in trying to convince” her not to have an epidural is informative of the limited power some women perceive that they have in labor. This is particularly so when we consider that Louise is in the midst of labor, is a patient in a hospital, and has no other way to access pain relief other than via her midwife, who has thus far refused her requests. She felt disempowered, hence implored her partner to strengthen her claim.

## Discussion

Many women are committed to the idea of a ‘drug free’ vaginal birth – because they consider this is the best type of birth for their baby as espoused by various authors [23, 54, 55]. In some cases when women encounter pain so severe that it exceeds their expectations and ask for pain relief, it appears that it may be denied by her caregivers. This denial of pain relief appears to be particularly traumatising to women when their expectations were that they would not likely need it. This study, while not designed to, unexpectedly revealed that some nulliparous women’s experiences of pain in labor left them traumatised for a number of reasons and this is likely contributed to by the existence of birth dissonance.

Part of the trauma experienced by these nulliparous women is that they perceive an expectation that it is better to aim for a natural birth, because women’s bodies are designed to do this and therefore they can and should [6, 23, 26]. Advocates of a ‘natural birth’ without pain relief argue that it makes better mothers consequent to them experiencing the transformative effects of natural birth. Leap and Anderson [23] are vocal proponents of childbirth without pain relief, arguing that women who undergo birth with epidural anaesthesia become ‘passive and biddable’ (p. 28) as mothers, because that is how they have labored.

The study findings presented here challenge this broadly promulgated assumption that pain in labor as part of a ‘natural birth’ is an essential element of the process of the ‘rendering’ of a mother. Women’s choices for pain relief in labor need to be respected, regardless of others’ opinions about an apparent need to experience pain to become a mother at one extreme, through to freely promoting routine use of one or more of the numerous forms of pharmacological or non-pharmacological analgesia. The mismatch that women in our study had between what they expected versus their lived experience appeared to result in a dissonance. This particular form of dissonance seems to arise in women post-birth as a result of the expectations that they construct prior to birth regarding the birth experience and their bodily ability to manage that pain without pain relief, and ultimately, needing to use pain relief to manage that pain. For women in this study who asked for pain relief and had this request denied or deferred, their distress appeared to be greater than in/for women who had their pain relief request met.

### The development of birth dissonance

The discrepancy between expectation and reality of birth has not previously been distilled into a single term, but rather described as a ‘mismatch between expectation and reality’ [36, 44–46, 56]. To address this, we have developed the term ‘birth dissonance’. This is a particular type of dissonance experienced by women post-birth, and leads them to question their abilities not just as women, but also as mothers. This is in part because women are told that labor is part of the cultural event that is ‘preparation’ for motherhood. Birth is therefore, not just about having a baby – it is also about being transformed from woman to mother by birth itself [57]. When it does not go as planned, however, this can contribute to trauma [32].

Our findings suggest that when women’s pain was managed as and when they requested, they were less likely to develop post-birth trauma type symptoms *even if* they experienced unexpected intervention. This may be because they felt that they had at least a degree of control over some aspect of their birth experience, when other elements of it had deviated from their original plans. This study did not investigate why interventions were instigated, but rather focused on pain relief requests and how these requests were responded to by their carers. These results suggest that, despite their low-risk healthy status, few of the women in this study were realistically prepared for the actual experience of childbirth. This includes them not being aware of the possibility of interventions and pain and having pain relief deferred. It also suggests that within this setting, improvements might be made by medical staff and midwives as explained below, to better respond to women’s requests during labor.

Midwives, as the primary caregivers during labour and birth in many countries, bring their own philosophies and beliefs about the use or avoidance of interventions, including analgesia, to the birthing room. They are well placed to work ‘with woman’ to navigate the multitude of ways that pregnancy, labor and birth may be experienced. This includes providing comprehensive pre-birth education that more accurately reflects the many ways that labour and birth may deviate from the expectations that they may have developed from the various sources to which they have been exposed while pregnant.

### Limitations of the study

This study was a single site study conducted over a period of several years. The findings here are representative of the culture of that birthing unit and the birthing experience that women are having in that unit. Each birthing unit culture varies, and other studies in other units are required for comparative purposes. A larger study involving multiple interviewers and double coding of themes may be instructive in relation to other themes or reasons for post-birth trauma appearing in women post-birth. Two women did not complete all three interviews, but did complete two of the three. Only women who spoke English were able to be included owing to no interpreters being available for interviews in the study budget.

The researchers have had varied experiences of birth themselves, and bring their own experiences to this study. Two researchers have children which they birthed, one researcher has two children who are adopted; one researcher has worked extensively with birthing women but does not have children of her own. We acknowledge that these experiences impact the interpretation of the data.

## Conclusion

This study shows that even women with low-risk birthing profiles who choose to give birth in a hospital may have to engage with higher levels of technology, intervention and pain relief than that which they expected pre-birth. It also suggests that when these women request pain relief in birth and that request is not responded to as they had anticipated, they may be more likely to develop post-birth trauma. For women who asked for pain relief and had this request denied or deferred, their distress appeared to be greater and resulted in a particular form of trauma – namely birth dissonance. We are compelled to ask whether this trauma could be avoided by the use of improved pre-birth education for women about how painful labor is likely to be. Secondly, that education pre-birth needs to include a detailed explanation of the utility of pain relief, including the results of a recent Cochrane systematic review which shows that it does not lead to increased interventions. Thirdly more egalitarian decision-making during labor, and finally delivering upon women requests for pain relief in labor, at the time that they ask for it.[53]

### Supplementary Information

**Additional file 1.** Attachment A - Interview schedule for women having their first baby (Pre-Birth/Post Birth Interview).

**Additional  file 2.** Particpants were advised in the consent process that their data would be anonymised, and be used to create a thesis and may be used in publications.

## Data Availability

The data that support the findings of this study are available on request from the corresponding author ES. The data are not publicly available due to them containing information that could compromise research participant privacy/consent as given by participants and would require approval prior to release from the approving Human Research Ethics Committee.

## Abbreviations

DTM: Deep Tissue Massage
TENS: Transcutaneous Electrical Nerve Stimulation

## Footnotes

1 For women that appeared to be experience trauma type symptoms, a referral process had been set up in the ethics approval documents and this was followed for these women. This included follow up with the author’s Supervisor, referral to the birthing woman’s GP or other relevant service (in Australia this is BeyondBlue) or for the woman to seek earlier follow up with their midwife/community midwife for assessment and referral to other local services or whichever they preferred.
