# Supplementary material for: Women’s expectations about birth, requests for pain relief in labor and the subsequent development of birth dissonance and trauma
Source: BMC Pregnancy Childbirth. 2023 Nov 9;23:777. doi: 10.1186/s12884-023-06066-7 (PMC10633977; doi:10.1186/s12884-023-06066-7)
Supplement: Supplementary file 1 — Additional file 1. Attachment A - Interview schedule for women having their first baby (Pre-Birth/Post Birth Interview). [file 12884_2023_6066_MOESM1_ESM.docx]

**Attachment A - Interview Schedule for Women Having their First Baby**

**(Pre-Birth/Post Birth Interview)**

**Logos removed**

**Semi-Structured Interview Schedule – Interview I for Women Having Their First Baby**

**Project Background – to be explained by researcher.**

This research is looking at what happens to a woman’s request/s for pain relief in labour.  This research project seeks to examine:

o   what expectations a group of women have about pain relief before labour

o   what happens when women request pain relief in labour and how these requests are responded to;

o   how women think and feel about having to change their plans for pain relief treatment – if they did.

This research could be considered important because it has been suggested that there are two main models of thinking that guide treatment  –  one being a medical/technical approach which uses medical technology and other instruments to manage labour as opposed to an natural/organic model which relies on the resources of the body to manage pain and delivery in vaginal births.  Given these two models, and the commitment that carers may or may not have to each model and the difference between these models, I am trying to find what happens in labour when women request pain relief.

*This project aims therefore are to determine:*

o   How a group of women form expectations regarding pain and pain relief during labour, prior to giving birth

o   What expectations a group of women had about pain and pain relief, prior to the process of labour

o   When women make a request for pain relief during labour, how their request is responded to  - is it as the woman making the request hopes and expects

o   When women require different types of pain relief to that which they envisaged prior to birth, how they adjust to the variation from their original birth plan.

Process (to be explained by researcher):

This interview process involves two parts.  Part one is a data collection section.  The second part is a semi-structured interview that will take about an hour.  There are no right or wrong answers. Some questions are written down, but other questions will arise during the interview/conversation. If you feel uncomfortable at any time during the interview you should say so and we will move onto the next question.

**Firstly, you will be asked to fill in a brief survey (the data collection section).  This could be helpful in terms of comparing the experiences of women in the study.**

**Data Collection – Interview I – Prior to Birth (approx 36 weeks gestation)**

**Demographic Information**

**(Survey Items)**

1. What is your age at this time: _________years

2. What is you highest level of education? (please tick)

Primary  Secondary  Tertiary  Bachelors Degree Post Graduate Qualification

PhD

3. What is your usual occupation? ______________________

4. Are you:  in a domestic partnership  single   in a relationship? (circle)

4b    *Ask if in a domestic partnership*: what is your partner’s highest level of education?

Primary  Secondary  Tertiary  Bachelors Degree Post Graduate Qualification

PhD

4c    *Ask if in a domestic partnership*: what is your partner’s occupation?

5. Religion (if any): _________________

6. How do you self identify (in terms of ethnicity)?____________________

7. What is your postcode?______________

8. Have you been seeing a GP i.e your regular GP throughout your pregnancy? (circle below)

Yes   No   Been seeing a number of GPs but had regular GP care

9. Stage of Pregnancy at which you first attended XXXXXXXXXX?

_________weeks.

10. Number of weeks gestation at time of interview? _________weeks

11. How long do you think your labour will be?

□    1 – 3 hours

□ 4 – 7 hours

□ 8 – 11 hours

□ 12 – 15 hours

□ 16 – 19 hours

□ 20 – 23 hours

□ 24 hours or longer

12. What pain relief methods will you consider using for your labour?  (tick all that apply or arise during conversation)

□ Meditation

□ Hypnosis

□ Warm water

□ Movement

□ Deep Tissue Massage

□ TENS

□ Aromatherapy

□ Nitrous Oxide

□ Pethidine via intramuscular injection

□ Epidural

□ Spinal block

□ Other

13. Why have you included these methods?

**__________________________________________________________________________________________________________________________________________________________________________________________________________________________________________________________________________________________________________________________________________________________________________________________________________________**

14. Are there any types of pain relief that you would not consider using? Yes/ No (tick below which ones were excluded if any that arise during conversation)

□ Meditation

□ Hypnosis

□ Warm water

□ Movement

□ Deep Tissue Massage

□ TENS

□ Aromatherapy

□ Nitrous Oxide

□ Pethidine via intramuscular injection

□ Epidural

□ Spinal block

□ Other

15. Why did you exclude these?

______________________________________________________________________________________________________________________________________

___________________________________________________________________

16. What does ‘pain relief’ mean to you?

□ Reduction of pain to make it manageable, but it can still be clearly felt

□ Reduction of pain such that it can barely be felt but it is not absent

□ Absence of pain – no pain at all

□ Other

______________________________________________________________________________________________________________________________________

__________________________________________________________________

17. If you use a type of pain relief,  and it does not relieve your pain, what will you do? (Prompts below if required)

□ Asked for a different option immediately?

□ Waited to see if you could continue in your labour without a different pain relief intervention?

□ Determined to wait for a period of time and then asked for a different intervention?

□ Other course of action

______________________________________________________________________________________________________________________________________

___________________________________________________________________

18. Are you worried about the pain you may feel in labour? Yes/ No (please circle one)

If yes - why?

______________________________________________________________________________________________________________________________________

If no - why not?

______________________________________________________________________________________________________________________________________

___________________________________________________________________

19. What do you expect the health care staff looking after you to do about your pain while you are in labour?

______________________________________________________________________________________________________________________________________

20. Why do you think you expect these things?

______________________________________________________________________________________________________________________________________

___________________________________________________________________

**Data Collection - Semi Structured Interview – Probe questions.**

1. How would you describe your approach to pain management in labour?

2. Do you expect labour to be painful?

3. Why or why not?

4. If you hope for a natural birth – why do you hope for this?

5. Where have you or will you get information about pain and pain relief in labour?

6. Have you talked to other women who have told you about their births?

7. What did you think about what other women have told you? Do they provide you with comfort or do they make you worried about labour?

8. If your pain becomes severe, what will you do?

9. Describe what you consider to be the best birth experience possible.

10. If you have less pain than you expected, how do you think you will feel about the labour experience?

11. If you have more pain than you expected, how do you think you will feel about the labour experience?

12. Is there anything else you would like to add?
