# Supplementary material for: Women’s expectations about birth, requests for pain relief in labor and the subsequent development of birth dissonance and trauma
Source: BMC Pregnancy Childbirth. 2023 Nov 9;23:777. doi: 10.1186/s12884-023-06066-7 (PMC10633977; doi:10.1186/s12884-023-06066-7)
Supplement: Supplementary file 2 — Additional file 2. Particpants were advised in the consent process that their data would be anonymised, and be used to create a thesis and may be used in publications. [file 12884_2023_6066_MOESM2_ESM.pdf]

Participant Information Sheet/Consent Form
Health/Social Science Research - *Adult providing own consent*
(Women expecting their first baby)

[redacted]

**Title**	***The Labour of Pain - Pain Management in Labour***
**Short Title**	***The Labour of Pain***
**Protocol Number**	**15297A**
**Project Sponsor**	***Monash University***
**Coordinating Principal Investigator/ Principal Investigator**	***Professor Christine East and Associate Professor Andrea Whittaker***
**Associate Investigator(s)** ***(if required by institution)***	***Ms Liz Sutton – Monash University, Assoc Professor Bev Thiele – Murdoch Dr Evangelyn Malkoutzis –*** [redacted]
**Location** ***(where CPI/PI will recruit)***	[redacted]

Part 1 What does my participation involve?

03 Introduction

You are invited to take part in this research project, which is called *The Labour of Pain – Pain Management in Labour*. You have been invited because you meet the selection criteria for the study which are that you are having your first baby and are not planning an operative delivery and have not requested a scheduled epidural prior to admission or upon your admission. You are regarded as having a low risk pregnancy and you speak English.

The research team have not been provided with your contact details at any stage.

This Participant Information Sheet/Consent Form tells you about the research project. It explains the processes involved with taking part. Knowing what is involved will help you decide if you want to take part in the research.

Please read this information carefully. Ask questions about anything that you don't understand or want to know more about. Before deciding whether or not to take part, you might want to talk about it with a relative, friend or local health worker.

Participation in this research is voluntary. If you don't wish to take part, you don't have to.

If you decide you want to take part in the research project, you will be asked to sign the consent section. By signing it you are telling us that you:

- Understand what you have read
- Consent to take part in the research project
- Consent to be involved in the research described

• Consent to the use of your personal and health information as described.

You will be given a copy of this Participant Information and Consent Form to keep.

2 What is the purpose of this research?

The purpose of this study is to find out:
How a group of women (25) form expectations about pain and pain relief in labour
What expectations a group of women (25) have about pain relief prior to labour
What occurs when these women request pain relief in the midst of labour and how these requests are responded to; and
How these women feel about changing their plans for pain relief treatment – if they did.

This research could be considered important because it has been suggested that there are two main ways that people think about how they will manage pain labour. One of these is the medical/technical method which uses medical technology and other instruments to manage labour and labour pain. The other is the natural/organic model which relies on the resources of the body to manage labour and labour pain [redacted] **vaginal births.** Given these two approaches, and the commitment that carers may or may not have to them, and the difference between these approaches, we are trying to find out what happens in labour when women request pain relief.

This study is being conducted by Professor Christine East – Professor Midwifery and Co-Director of Maternity Services at [redacted]. She is being assisted by Liz Sutton – a student at Monash University studying for a PhD – this is a study that results in the writing of a thesis – like a 300 page book. If you have any queries, please direct them to Liz in the first instance. Her details are below.

Ms Liz Sutton
PhD Candidate
School of Political and Social Inquiry
Faculty of Arts
Monash University – Clayton
Phone : [redacted]
email :[redacted]

No funding is attached to this research – it is primarily for the purpose of learning about pain management in labour.

The results of this research will be used by the researcher Liz Sutton to obtain a Doctor of Philosophy degree.

This research has been initiated by the researcher, Liz Sutton.

3 What does participation in this research involve?

The project involves three in depth interviews – which will be audiotaped for reference purposes. The three interviews occur at the following intervals: one month prior to the expected delivery date, one to two weeks after delivery of your baby – within the first week would be ideal; and a final one six months after delivery. You have been chosen as a potential participant because you are having your first baby and are not planning an operative delivery and have not requested a scheduled epidural prior to admission or upon your admission. You are regarded as having a low risk pregnancy and you speak English.

Each interview should take about one hour. The survey should take about ten minutes. Surveys will be collected at the end of the interview session. In total you should expect your involvement in this research project to take up three and half hours over a total time period of about seven months, in addition to discussing this participant information and consent form.

Please note: All participants in this research project are being recruited via an advertising/recruitment poster or through direct contact with me, or are referred to me by either other participants or word of mouth. The research team has not had access to your contact details or other identifying information unless you have chosen to provide me with this information directly. Once provided, your contact details will be safeguarded by researchers following Monash University Protocols for the safeguarding of information and only the Monash University research team comprising Liz Sutton, Associate Professor Andrea Whittaker and Professor Christine East, will have access to them

Why were you chosen for this research?

This research project requires 3 in-depth interviews to be conducted with 25 women. For this study, we are looking only at women who:

- are having their first baby
- are planning to have a ‘non-operative’ birth
- are regarded as having a low risk pregnancy
- are booked to deliver in a hospital setting
- do not have a pre-planned epidural
- are aged 18 – 34
- identify as Australian and speak English.

There will be no cost to you to participate in this research, aside from the time that you will need to set aside for the questionnaires and interviews. Participants are asked to volunteer their time as funds for payment of participant time are not available.

During the research and data collection process Liz will be meeting with her PhD supervisory team (at a minimum one of her academic supervisors) every fortnight. This research has been approved by [redacted] Ethics Committee and Liz is required to report on progress to the

University through her supervisor and at a review of her candidature in February 2016. Issues that arise during the research will be discussed with both Professor Christine East and Associate Professor Andrea Whittaker who are Liz's main academic supervisors for this research.

It is a requirement that participants can speak English. In relation to the venue for the interviews, you may choose where you wish to be interviewed, with suggested locations being on site at the hospital in an assigned room, at a local café that is large enough to enable us to have a confidential conversation, or at a local community space that is public such as a community centre or library with meeting rooms.

If you decide to take part in the research project, you will first be given a questionnaire asking about whether you meet the inclusion criteria for the project. The questions help me to determine if you are able to participate in the study. They ask about whether you have had a labour before, what sort of birth you are planning and if you have any illnesses or conditions that affect your pregnancy. (see Attached Screening Questions)

If the screening questionnaire shows that you meet the requirements, then you will be able to start the research project. If the screening questionnaire shows that you cannot be in the research project, the research coordinator will discuss other options with you.

This research project has been designed to make sure the researchers interpret the results in a fair and appropriate way and avoids study doctors or participants jumping to conclusions. This is one of the key reasons that the research involves interviewing a number of groups such as doctors, midwives, anaesthetists and women who are expecting their first baby and go on to deliver. Data will be analysed using accepted methods for the analysis of qualitative data, and academic progress is reported to Monash University through Associate Professor Andrea Whittaker and through student milestones that Liz needs to meet.

There are no costs associated with participating in this research project, nor will you be paid.
However, you may be reimbursed for any reasonable travel, parking, meals and other expenses associated with the research project visit.

4 Other relevant information about the research project

The number of people taking part in this project will be approximately 40 overall. The research design includes the use of computer programs that are designed to detect themes and patterns in the things that people tell us in the course of the research. This will help make the analysis of what people have said easier to understand and write about.

The research relies on four groups of people agreeing to participate – these are women who are having their first baby ([redacted] with no prior experience of labour; midwives

working at [redacted] in the maternity units; [redacted] providing anaesthetics to women in labour; and Obstetricians working at [redacted] providing obstetric services to women delivering their babies at these sites.

All of the field research is being conducted at sites that are managed by [redacted] The researchers engaged in the project come from a number of different organisations, which are:
Monash University
[redacted]
Murdoch University

The research design relies on the two sites for field research to be conducted – which are [redacted]

This research came about because Liz Sutton – one of the researchers, completed a Masters Degree in which she looked at how labour pain appeared to be treated differently to other types of acute pain. Liz was interested in the philosophical approaches to the management of labour pain and wanted to better understand the experience that women had of labour, and how their requests for pain relief were managed. This is because the literature that she looked at for her Masters thesis indicated that many women hoped for one type of birth, and often their experience didn't match what they had hoped for.

5 Do I have to take part in this research project?

Participation in any research project is voluntary. If you do not wish to take part, you do not have to. If you decide to take part and later change your mind, you are free to withdraw from the project at any stage up to the point of publication of the results in a thesis – around late 2019.

If you do decide to take part, you will be given this Participant Information and Consent Form to sign and you will be given a copy to keep.

Your decision whether to take part or not to take part, or to take part and then withdraw, will not affect your routine care, your relationship with professional staff or your relationship with [redacted].

6 What are the possible benefits of taking part?

We cannot guarantee or promise that you will receive any benefits from this research; however, possible benefits may include the provision of an opportunity for you to talk about your birth experience. Some women wish to 'de-brief' about their birth experience and research suggests that this is an area that women may benefit from post delivery.

7 What are the possible risks and disadvantages of taking part?

Possible Risks

Birth is a highly personal experience. Prior to the birth of your baby you may be willing to participate in this study, however the idea of reflecting on your birth experience afterwards may be traumatising for you.

Evidence suggests that some women can develop Post Traumatic Stress Disorder following childbirth, for a number of reasons. Revisiting the birth experience may exacerbate any negative feelings you have about the birth, and this may mean that you experience psychological discomfort.

If, prior to, during, or after the birth of your baby, you feel that the experience of talking about the birth would be traumatising, you should feel free to withdraw from the study. You can withdraw from the study at any time up to the time of results being published in a thesis I will be writing. This will occur around late 2019.

You may feel that some of the questions we ask are stressful or upsetting. If you do not wish to answer a question, you may skip it and go to the next question, or you may stop immediately. If you become upset or distressed as a result of your participation in the research project, the research team will be able to arrange for counselling or other appropriate support. Any counselling or support will be provided by qualified staff who are not members of the research team. This counselling will be provided free of charge.

8 What if I withdraw from this research project?

If you do consent to participate, you may withdraw at any time. If you decide to withdraw from the project, please notify a member of the research team before you withdraw. A member of the research team will inform you if there are any special requirements linked to withdrawing. If you do withdraw, you will be asked to complete and sign a 'Withdrawal of Consent' form; this will be provided to you by the research team.

If you decide to leave the research project, the researchers will not collect additional personal information from you, although personal information already collected will be retained to ensure that the results of the research project can be measured properly and to comply with law. You should be aware that data collected up to the time you withdraw will form part of the research project results. If you do not want your data to be included, you must tell the researchers when you withdraw from the research project.

9 Could this research project be stopped unexpectedly?

This research project may be stopped unexpectedly for a variety of reasons. These may include reasons such as researcher illness, withdrawl of approval to conduct the study at the stated sites; other issues relating to the impacts of the study on both birthing women and personnel employed at the two health sites.

10 What happens when the research project ends?

When the research project ends, should you wish to, you will be able to obtain a copy of the completed thesis by emailing Liz at: [redacted] We anticipate that this will be late 2019. Your individual interview and data obtained from it, can be provided to you once the interview process with you is complete and the data as a complete set, has been analysed – we anticipate that this will be mid to late 2018.

Part 2 How is the research project being conducted?

11 What will happen to information about me?

By signing the consent form you consent to the research team collecting and using personal information about you for the research project. Any information obtained in connection with this research project that can identify you will remain confidential. Your healthcare team will not have access to it, and upon commencement each participant will be assigned a unique identifier. Once assigned the list of names and unique identifiers will be kept at Monash University, aware from the data set. Only the research team will have access to it and it will be kept in a locked filing cabinet at Monash University. Your information will only be used for the purpose of this research project and it will only be disclosed with your permission, except as required by law.

The personal information that the research team collect and use is the information that you provide in interviews, your completed surveys and comments that you provide in the in depth interviews.

Information about you may be obtained from your health records held at this and other health organisations for the purpose of this research. By signing the consent form you agree to the research team accessing health records if they are relevant to your participation in this research project.

It may be useful for the research team to look at your medical file to determine how your labour progressed and how your pain was managed. This information will also be treated confidentially and will not be used for any other purpose other than cross referencing your interview and the verbal account of your labour with the experience of it as recorded in your medical file.

It is anticipated that the results of this research project will be published and/or presented in a variety of forums. In any publication and/or presentation, information will be provided in such a way that you cannot be identified, except with your express permission. Where case studies are provided, names and ages will be changed. Where data is used for publication, the data will not be presented alongside of potentially identifying information. Unique identifiers will be used to differentiate cases where multiple cases are being presented, rather than pseudonyms to protect confidentiality.

In accordance with relevant Australian and Victorian privacy and other relevant laws, you have the right to request access to the information about you that is collected and stored by the research team. You also have the right to request that any information with which you disagree, be corrected. Please inform the research team member named at the end of this document if you would like to access your information.

Any information obtained for the purpose of this research project that can identify you will be treated as confidential and securely stored for 7 years following completion of the study. It will be disclosed only with your permission, or as required by law.

12 Complaints and compensation

If you have a complaint or a concern about the clinical care that you have received, then please contact the Consumer Liaison Officer of your healthcare provider. Contact details are below:

Consumer Liaison Officer Contact Numbers:

[redacted]

If you suffer any distress or psychological injury as a result of this project, you should contact the research team as soon as possible. You will be assisted with arranging appropriate treatment and support

13 Who is organising and funding the research?

This research project is being conducted by Liz Sutton with supervision from Professor Christine East and Associate Professor Andrea Whittaker. Liz is a student of Monash University. Funding has not been provided for this research by any agency/company.

It is not likely that [redacted] or Monash University will benefit financially from this research, as it is unlikely to assist them in any commercial enterprise.

You will not benefit financially from your involvement in this research project even if, for example, knowledge acquired from your information proves to be of commercial value to [redacted] or Monash University.

In addition, if knowledge acquired through this research leads to discoveries that are of commercial value to [redacted] and Monash University the researchers or their institutions, there will be no financial benefit to you or your family from these discoveries.

No member of the research team will receive a personal financial benefit from your involvement in this research project (other than their ordinary wages).

14 Who has reviewed the research project?

All research in Australia involving humans is reviewed by an independent group of people called a Human Research Ethics Committee (HREC).
The ethical aspects of this research project have been approved by [redacted] and subsequently the HREC of Monash University.
This project will be carried out according to the National Statement on Ethical Conduct in Human Research (2007). This statement has been developed to protect the interests of people who agree to participate in human research studies.

15 Further information and who to contact

The person you may need to contact will depend on the nature of your query. If you want any further information concerning this project or if you have any problems which may be related to your involvement in the project, you can contact the researcher on 0421 582 350 or email at easut3@student.monash.edu – or any of the following people:

Research contact person

Name	Professor Christine East
Position	[redacted]
Email	

Name	Associate Professor Andrea Whittaker
Position	Associate Investigator – School of Political and Social Inquiry Monash University
Telephone	[redacted]
Email	[redacted]

Name	Dr Evangelyn Malkoutzis
Position	[redacted]
Telephone	03 9594 3283
Email	[redacted]

For matters relating to research at the site at which you are participating, the details of the local site complaints person are:

Complaints contact person

Name	Ms Deborah Dell
Position	Manager, Human Research Ethics Committee
Telephone	[redacted]
